# Low Voltage Activation of KCa1.1 Current by Cav3-KCa1.1 Complexes

**DOI:** 10.1371/journal.pone.0061844

**Published:** 2013-04-23

**Authors:** Renata Rehak, Theodore M. Bartoletti, Jordan D. T. Engbers, Geza Berecki, Ray W. Turner, Gerald W. Zamponi

**Affiliations:** 1 Department of Physiology and Pharmacology, Hotchkiss Brain Institute, University of Calgary, Calgary, Canada; 2 Department of Cell Biology and Anatomy, Hotchkiss Brain Institute, University of Calgary, Calgary, Canada; University of Houston, United States of America

## Abstract

Calcium-activated potassium channels of the KCa1.1 class are known to regulate repolarization of action potential discharge through a molecular association with high voltage-activated calcium channels. The current study examined the potential for low voltage-activated Cav3 (T-type) calcium channels to interact with KCa1.1 when expressed in tsA-201 cells and in rat medial vestibular neurons (MVN) *in vitro*. Expression of the channel α-subunits alone in tsA-201 cells was sufficient to enable Cav3 activation of KCa1.1 current. Cav3 calcium influx induced a 50 mV negative shift in KCa1.1 voltage for activation, an interaction that was blocked by Cav3 or KCa1.1 channel blockers, or high internal EGTA. Cav3 and KCa1.1 channels coimmunoprecipitated from lysates of either tsA-201 cells or rat brain, with Cav3 channels associating with the transmembrane S0 segment of the KCa1.1 N-terminus. KCa1.1 channel activation was closely aligned with Cav3 calcium conductance in that KCa1.1 current shared the same low voltage dependence of Cav3 activation, and was blocked by voltage-dependent inactivation of Cav3 channels or by coexpressing a non calcium-conducting Cav3 channel pore mutant. The Cav3-KCa1.1 interaction was found to function highly effectively in a subset of MVN neurons by activating near –50 mV to contribute to spike repolarization and gain of firing. Modelling data indicate that multiple neighboring Cav3-KCa1.1 complexes must act cooperatively to raise calcium to sufficiently high levels to permit KCa1.1 activation. Together the results identify a novel Cav3-KCa1.1 signaling complex where Cav3-mediated calcium entry enables KCa1.1 activation over a wide range of membrane potentials according to the unique voltage profile of Cav3 calcium channels, greatly extending the roles for KCa1.1 potassium channels in controlling membrane excitability.

## Introduction

Calcium-activated potassium channels are expressed in central neurons to control membrane excitability following activation by calcium influx through voltage- or ligand-gated channels [1]–[4]. Of the known isoforms of calcium-dependent potassium channels, the “big conductance” (BK, KCa1.1) channel plays a key role in controlling action potential repolarization and a fast afterhyperpolarization in numerous cell types [1], [5]–[9]. The calcium influx required to activate KCa1.1 channels can derive from high voltage-activated (HVA) calcium channel conductance by virtue of a molecular association between KCa1.1 and HVA calcium channels [6], [10]–[14]. This is important in activating KCa1.1 channels according to the distribution and voltage range of specific HVA calcium channel isoforms [2], [15]–[18]. Given that the principal membrane depolarization large enough to traverse the voltage range for HVA calcium channels is an action potential, KCa1.1 activation is normally thought to be restricted to suprathreshold events. An exception is the role played by the Cav1.3 class of HVA calcium channel activated in the −50 mV range to control the rate of firing in chromaffin cells [9]–[10], [19]. Much less is known about the potential for KCa1.1 current to be activated by Cav3 channels that operate in an even lower voltage range to regulate membrane excitability and spike output in neurons.

Calcium influx in the low voltage range can be mediated by Cav3 T-type calcium channels, with an established ability for Cav3 calcium influx to activate either small or intermediate conductance potassium channels [20]–[21]. A potential interaction between Cav3 and KCa1.1 channels is supported by a report of coimmunoprecipitation between Cav3.2 and KCa1.1 proteins from mouse brain lysates [22], and at least a functional coupling between Ni^2+^-sensitive calcium influx and KCa1.1-mediated AHPs in neurons of the medial vestibular nucleus (MVN) [23]. However, direct biophysical tests for a Cav3-KCa1.1 interaction have not been reported, and the molecular sites that would support an association between Cav3 and KCa1.1 channels have not been determined.

The current study provides biochemical and biophysical evidence for a novel signaling complex between Cav3 and KCa1.1 channels in both a heterologous expression system and in some MVN cells. Moreover, the Cav3-mediated activation of KCa1.1 confers the unique voltage profile of Cav3 calcium channels upon KCa1.1 current, extending the potential voltage range for KCa1.1 activation to both the low and high voltage realms of membrane potential deflections. The widespread expression of these channel subtypes suggest that a Cav3 mediated KCa1.1 regulation may be relevant to the control of membrane excitability in a wide range of cell types.

## Methods

### Ethics Approval

Experimental procedures for this study were approved by the University of Calgary Animal care committee. Timed-pregnant Sprague-Dawley rat pups were obtained from Charles River (Saint-Constant, PQ) and maintained according to guidelines of the Canadian Council for Animal Care. Tissue slices were prepared from rats of postnatal day 12–16. All chemicals were obtained from Sigma (St. Louis, MO) unless otherwise noted.

### Molecular Biology

Wild-type human Ca_v_3.2 cDNA was a gift from T. Snutch (University of British Columbia) and wild-type human KCa1.1 cDNA a gift from A. Braun (University of Calgary). To create a Cav3.2 pore mutant (Ca_v_3.2^pm^), site-directed mutagenesis (Stratagene now Agilent, Mississauga, ON) was performed using a 1.9-Kb fragment of Ca_v_3.2 flanking the domain I pore region, followed by sequencing and subcloning into full length Ca_v_3.2 cDNA. The Cav3.2 full length HA-tagged clone was generated from a Cav3.2 HA GFP tagged clone provided by E. Bourinet (IGF, Montpellier). The KCa1.1 full length myc-tagged clone used for luminometry was constructed by first generating a 5′- myc-tagged KCa1.1 N-terminus by PCR, followed by sequencing and subcloning unto full length KCa1.1. KCa1.1ΔC-term, KCa1.1 NSO, KCa1.1 aNSO, KCa1.1 N-term, and KCa1.1 C-term were all generated by PCR, followed by sequencing and subcloning into a mammalian expression vector, pCDNA3.1- (Invitrogen, Burlington, ON).

### Transient Transfection

Ca_v_3.2 and KCa1.1 constructs were transfected into tsA-201 cells using the calcium phosphate method. For coimmunoprecipitation and electrophysiology, 5 µg of channel cDNA was used, with enhanced green fluorescent protein (GFP) cDNA (1 µg) added to identify transfected cells for electrophysiology. For coexpression studies, 5 µg of each channel cDNA was co-transfected, and empty vector was used as control cDNA. Transfected cells were incubated at 37°C (5% CO_2_) for 24 hr and then transferred to a 29°C incubator (5% CO_2_) for 2–3 days prior to recording.

### Cell Surface Luminometry Assay

Cultured tsA-201 cells were transiently transfected with myc-KCa1.1 with or without Ca_v_3.2 constructs. Cell surface luminometry assay was performed as previously described [24]. Assay was performed using anti-myc primary antibody (1∶2000, Roche, Indianapolis, PA) and probed with HRP-conjugated sheep anti-mouse antibody (1∶1500, GE Healthcare, Baie d’Urfe, QC). The signal intensity from permeabilized cells was used as a measure of total cellular channel expression, and from non-permeabilized cells as a measure of channel surface expression (*n* = 6).

### tsA-201 Electrophysiology

Whole-cell patch clamp recordings from tsA-201 cells were performed at room temperature using an Axopatch 200B amplifier (Molecular Devices, Union City, CA) and pipettes of 3–6 MΩ resistance. The KCa1.1 recording solutions when recorded with or without coexpression of Cav3.2 contained (in mM): External: 120 NaCl, 3 NaHCO_3_, 4.2 KCl, 1.2 KH_2_PO_4_, 1.5 MgCl_2_, 10 Dextrose, 10 HEPES, 1.5 CaCl_2_ (pH 7.3). Internal: 140 KCl, 5 HEPES, 0.1 EGTA, 0.5 MgCl_2_, 5 ATP, 1 GTP (pH 7.3). The Ca_v_3.2 recording solutions contained (in mM): External: 142 CsCl, 2 CaCl_2_, 1 MgCl_2_, 10 HEPES, 10 Glucose (pH 7.4). Internal: 126.5 CsCH_3_SO_3_, 2 MgCl_2_, 11 EGTA, 10 HEPES (pH 7.4). All recordings in tsA-201 cells were conducted in the presence of external 1 mM 4-AP to reduce a small amplitude endogenous potassium current (data not shown) and leak subtracted offline in Clampfit 10.2 (Molecular Devices, Union City, CA).

Whole cell current-voltage relationships for Cav3.2 were acquired from a holding potential of −100 mV in 10 mV steps between −90 mV and 50 mV. Conductance plots were fitted with a modified Boltzmann equation (I = (1/(1+exp(-(V_a_-V)/S)))*(V-Erev)*G_max_, where ‘I’ is current, ‘V_a_’ is half-activation potential, ‘V’ is membrane potential, ‘E_rev_’ is reversal potential, S is the slope factor, and ‘Gmax’ is slope conductance. Steady-state inactivation curves of Cav3.2 current were acquired using preconditioning potentials (4 s) and a 150 ms test pulse to −30 mV, with data points fit using the Boltzmann equation (I/Imax = A2+ A1/(1+exp((V-Vh)/S)), where ‘I/Imax’ is normalized current, ‘A2’ is the non-inactivating fraction, ‘A1’ is inactivating fraction, ‘V’ is membrane potential, S is the slope factor, and ‘V_h_’ is half inactivation potential). KCa1.1 current was evoked in tsA-201 cells using a two pulse protocol (P1–P2) and most often measured as current density (peak amplitude normalized to cell capacitance, pA/pF) and expressed as the ratio between P2/P1.

### Slice Preparation

Rats were anesthetized with halothane and the brain removed in ice-cold artificial cerebrospinal fluid (aCSF) composed of (in mM): 125 NaCl, 3.25 KCl, 1.5 CaCl_2_, 1.5 MgCl_2_, 25 NaHCO_3_, and 25 D-glucose preoxygenated by carbogen (95% O_2_, 5% CO_2_) gas. Coronal slices (240 µm) of the pontine region and MVN were cut by vibratome in cold aCSF and slices transiently elevated to 34°C (60 min) before storing in carbogen-gased aCSF at room temperature. Slices were transferred to a recording chamber on a Zeiss Axioskop FS-2 microscope (Thornwood, NY) equipped with differential interference contrast optics and infrared light transmission, and maintained as a submerged preparation.

### Slice Electrophysiology

Whole-cell patch recordings were obtained in MVN cells at 32–34°C using a Multiclamp 700B amplifier (Molecular Devices, Union City, CA) with pipettes of 3–6 MΩ resistance and access resistance 8–15 MΩ (80–90% compensation in voltage clamp). The internal solution for voltage-clamp and current clamp recordings was (in mM): 140 KGluconate, 0.1 EGTA, 10 HEPES, and 2.5 MgCl_2,_ pH adjusted to 7.3 with KOH, with all step potentials adjusted for a calculated tip potential of 14 mV. Solutions containing 1 µM paxilline were shielded from light to prevent loss of activity. All recordings were performed in bath applied synaptic blockers picrotoxin (50 µM), DL-2-amino-5-phosphonopentanoic acid (DL-AP5, 25 µM), and 6,7-dinitroquinoxolinedione (DNQX, 10 µM, Tocris Cookson, Ellisville, MO). EGTA or BAPTA (tetrapotassium salt, Invitrogen, Burlington, ON) were dissolved in the electrolyte for cell dialysis.

Spike threshold was determined using a custom Matlab (Mathworks) script and was defined as the point where the velocity of voltage change exceeded five times the voltage standard deviation in the 50–100 ms preceding the action potential. The amplitude of the AHP was also analyzed with a custom Matlab script and was defined as the difference between the threshold voltages immediately preceding spike generation to the peak of the post-spike negative trough. Gain was determined by linear regression analysis in OriginPro 8.

### Tissue Slice Immunocytochemistry

Adult males (∼250 g) were deeply anesthetized using an overdose of inhaled isofluorane, perfused intracardially with 250 ml of 0.1 M phosphate buffer (PB, pH 7.4) followed by 100 ml 4% paraformaldehyde (PARA, pH 7.4) at room temperature. Brains were postfixed in 4% PARA at room temperature for 1 hr and overnight at 4°C. Free-floating sections (40–50 µm) were cut by vibratome and processed in a working solution of 3% normal donkey or horse serum (Jackson Immuno-Research, West Grove, PA), 0.2% TWEEN and 2% dimethylsulphoxide in PB. Primary antibodies were reacted for 48 hrs at 4°C, washed in working solution, and exposed to secondary antibodies for 4 hrs at room temperature.

Primary antibodies used for tissue immunocytochemistry were a rabbit polyclonal anti-Cav3.2 (1∶250) directed against amino acids 1195–1273 of the II-III linker (accession number # AF290213) [25], and a mouse monoclonal anti-Mslo (KCa1.1, clone L6/60) produced against fusion protein amino acids 690–1196 (NeuroMab, UC Davis). Secondary antibodies were goat anti-mouse AlexaFluor-488 and donkey anti-rabbit AlexaFluor-594 IgGs (1∶1000, Molecular Probes, Burlington, ON). Controls consisted of omission of primary antibodies. Images were obtained using a Zeiss LSM 510 confocal microscope and processed in Image J, Abobe Photoshop and Illustrator software, with processing restricted to producing extended images of multiple sections from confocal stacks, and contrast and brightness adjustments.

### Coimmunoprecipitation Assays

Rat brain tissue and tsA-201 cells were lysed in (in mM): 50 Tris, 150 NaCl, 1% NP-40, 0.5% NaDeoxycholate, 0.5% triton x-100, protease inhibitor cocktail, pH 7.5. Solubilised proteins were incubated with 2 µg of commercial polyclonal anti-Cav3.2 (H-300, sc-25691, Santa Cruz) developed against amino acids 2174–2353 of human Cav3.2, or 7 µl of rabbit polyclonal anti-Cav3.2 (as above) [25] and 30 µl of Protein A/G beads (GE Healthcare, Baie d’Urfe, QC). Western Blot analysis was performed using anti-myc (1∶1000, Roche, Indianapolis) and anti-KCa1.1 (1∶1000, clone L6/60, NeuroMab, Davis, CA) primary antibodies and probed with HRP-conjugated sheep anti-mouse antibody (1∶1500, GE Healthcare, Baie d’Urfe, QC). Two hours prior to lysis, plates were incubated in either DMEM (no serum) or DMEM (no serum). Lysis, coimmunoprecipitation, and Western Blot analysis followed as described above.

### Modeling

I_CaT_ activation
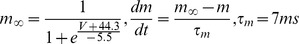



*I_CaT_* inactivation
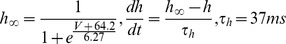



For all simulations, E_Ca_ = 40 mV. All simulations were constructed in Matlab R2007b using a fourth-order Runge-Kutta algorithm with a time step (dt) of 0.0001 ms. The maximum open probability for Cav3 channels was set to 0.25 [26]. To calculate calcium influx through the Cav3 channel, we assumed a single channel conductance of 1.7 pS (ĝ_CaT_  = 1.7 pS). To simulate multiple Cav3 channels, 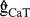 was multiplied by the number of desired channels. The number of incoming calcium ions (N_Ca_) was determined by:
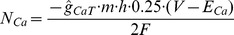
where F is Faraday’s constant (9.649 · 10^4^ coulombs/mol) [27].

### Calcium Diffusion Model

In order to determine K_Ca_ channel activation, calcium diffusion away from the Cav3 calcium source was modeled using 10 hemispherical compartments with calcium diffusion determined using the following explicit equation [27]:
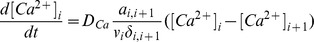
where D_Ca_ is the diffusion coefficient for calcium (0.220 µm^2^ms^–1^), *a_i,i+1_* is the surface area between the adjacent compartments, *v_i_* is the volume of the first compartment, *δ_i,i+1_* is the distance between compartments, and the term in the brackets represents the concentration gradient. The radius of the smallest compartment was 20 nm and the radius of each consecutive compartment was increased by 20 nm. The activation of the K_Ca_ channels was made to depend on the [Ca^2+^] of a particular compartment. Therefore, the effect of calcium on K_Ca_ channels could be observed for distances of up to 200 nm from the calcium source.

### Large Conductance Calcium-activated Potassium Channel (KCa1.1) Model

The parameters for voltage- and calcium-dependence of KCa1.1 channels previously described for Purkinje cells [28] were used to develop a model of the KCa1.1 channel. The equation for τ was adapted from a previous model of a calcium-activated potassium channel [29]. The calcium-dependence for V_1/2_ was adjusted to allow near maximal shift in V_1/2_ with 10 µM [Ca^2+^]_i_.

The relationship between the V_1/2_ of activation and calcium concentration (in µM) can be described by the equation:
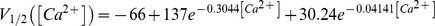



The maximum value of *p_open_* for a given voltage over all concentrations of calcium is given by the equation:
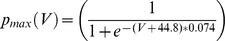



Using *p_max_* and the dissociation constant of calcium at a given voltage, *K_D_*_,_ the maximum *p_open_* for a given [Ca^2+^] is:
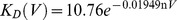


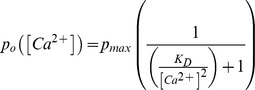



Finally, the activation rates and time constant, τ, are:
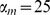


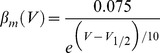


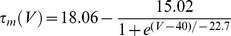



The differential equation to describe the activation variable of KCa1.1 channels, *m*, is therefore:
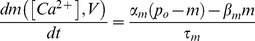



*Statistical Analysis -* Statistical analysis was conducted in OriginPro 8 by paired or unpaired student’s t-test, Wilcoxin signed ranks test or one-way analysis of variance (ANOVA) as appropriate. All error bars are ± S.E.M., with *P<0.05, **P<0.01, ***P<0.001.

## Results

### Cav3.2 Activation of KCa1.1 Current in the tsA-201 Expression System

**Figure 1:**
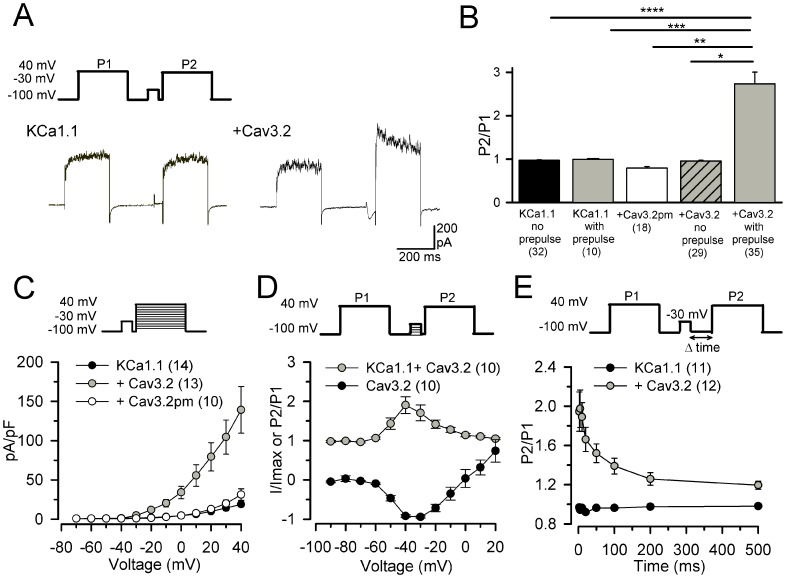
Cav3.2 channels increase KCa1.1 current by shifting activation to hyperpolarized potentials. Shown are whole-cell patch clamp recordings from tsA-201 cells transiently transfected with Cav3.2 or KCa1.1 cDNA. *A,* Representative KCa1.1 currents evoked with or without coexpression of Cav3.2 channels. KCa1.1 activation was tested by 250 ms steps to +40 mV (P1, P2) and the P2 pulse immediately preceded by a 50 ms test pulse to −30 mV (2 ms return to −100 mV) to maximally activate Cav3 current. *B,* Bar plots showing the fractional change (P2/P1) in KCa1.1 current elicited by the protocol in (*A*) and with Cav3.2 channels substituted with a Cav3.2 pore mutant (Ca_v_3.2^pm^) that does not conduct calcium. *C,* Current-voltage plots indicating that a pre-pulse to −30 mV to activate Cav3 current (see *inset*) significantly shifts KCa1.1 activation to more hyperpolarized potentials. KCa1.1 activation is unaltered when coexpressed with Ca_v_3.2pm. *D,* Plots of the P2/P1 ratio of KCa1.1 current evoked at +40 mV in a tsA-201 cell as a function of the voltage of a 50 ms pre-pulse command delivered in 10 mV increments from −90 mV to +20 mV (see *inset*). Note the close correspondence in voltage-dependence and magnitude of Cav3.2 and KCa1.1 currents. *E,* Time dependence of KCa1.1 activation from the effects of a pre-pulse to −30 mV (time 0) with test pulses to +40 mV (see *inset*) delivered at the indicated time intervals in cells with or without coexpression of Cav3.2 channels. Capacitance artefacts in (*A*) were digitally reduced for display purposes. Sample sizes are shown in brackets.

To examine the potential interaction between Cav3 and KCa1.1 channels, we transiently expressed cDNA for both channels in tsA-201 cells. Although the expression of ß subunits can modify KCa1.1 function [30]–[31], we expressed KCa1.1 cDNA in isolation to identify any specific interactions between Cav3 and KCa1.1 α-subunits. We also focused on the expression of Cav3.2 calcium channels given that calcium current coupled to generation of an AHP in MVN cells is sensitive to 100 µM Ni^2+^ [23], a concentration relatively selective for the Cav3.2 channel isoform [32]. In tsA-201 cells expressing KCa1.1 in isolation, a step command to +40 mV (250 ms) from a holding potential of −100 mV evoked a small amplitude non-inactivating outward current (**Fig. 1** ***A***). When KCa1.1 channels were coexpressed with Cav3.2 channels, a single pulse to +40 mV also produced little KCa1.1 current (data not shown). Therefore we used a two pulse protocol (P1, P2) with an interpulse interval of 250 ms in order to precede the P2 pulse with a 50 ms prepulse to −30 mV to maximally activate Cav3 current. This also allowed us to have an internal control (P1) to compare the amplitude of KCa1.1 current evoked by P2. Any change in outward current was then expressed as a ratio of P2/P1 current density or P2/P1 total current.

Control tests without a −30 mV prepulse indicated that the KCa1.1 current evoked by the P2 pulse was not significantly different from that elicited by P1 in cells either with Cav3.2 coexpression (P2/P1 = 0.96±0.2, *n* = 29, *P* = 0.926) or without Cav3.2 coexpression (P2/P1 = 0.97±0.01, *n* = 32, *P = *0.804) (**Fig. 1** ***B***). In contrast, preceding P2 with a −30 mV prepulse in cells coexpressing Cav3.2 and KCa1.1 evoked a 2.7±0.27 fold increase in the P2/P1 ratio (*n* = 35, *P<*0.001) (**Fig. 1** ***A and B***). Under these conditions, the KCa1.1 current activated on the P2 pulse typically exhibited a moderate and slow rate of decay (τ = 117.09±15.11 ms, 56.13±3.2% current remaining at 250 ms, *n* = 35) (**Fig. 1** ***A***). In contrast, co-expression of KCa1.1 with a Cav3.2 pore mutant (Cav3.2^pm^) that is incapable of conducting calcium ions blocked the increase in KCa1.1 current on the P2 pulse (P2/P1 = 0.80±0.04, *n* = 18, *P* = 0.390) (**Fig. 1** ***B***).

It was previously shown that the Cav1.3 (L-type) calcium channel can change the expression levels of KCa1.1 channels [33]–[34]. It is possible that co-expression of Cav3.2 channels could also increase KCa1.1 surface expression, changing the apparent availability of KCa1.1 channels for activation by calcium influx. To test for possible changes in KCa1.1 channel density at the membrane we performed a luminometry assay using a KCa1.1 clone with an external myc-tag. This allowed us to compare the relative level of membrane expression (non-permeabilized cells) to total KCa1.1 protein expression (permeabilized cells) for each condition. These tests established that there was no significant difference in KCa1.1 total protein expression levels in the absence or presence of Cav3.2 coexpression (*n* = 6, *P* = 0.62). There was also no significant difference in the percentage of KCa1.1 channels at the cell surface in the absence or presence of Cav3.2 coexpression (30.7±11.8% vs. 23.1±9.1%, *n* = 6; *P* = 0.394) (data not shown). Conversely, we tested whether KCa1.1 channels exerted any reciprocal regulation of Cav3.2 channel activity by recording Cav3.2 current in the presence of KCa1.1 channels when 11 mM EGTA internal was included to block KCa1.1 activation. These tests showed no significant effect of KCa1.1 channels on Cav3.2 current density or voltage-dependent properties (**Fig. S1**). The increase in KCa1.1 current in the presence of Cav3.2 calcium channels is then not due to an increase in KCa1.1 channel density or a reciprocal regulation of Cav3 channel properties.

### Cav3.2 Co-expression Shifts Activation of KCa1.1 to More Hyperpolarised Potentials

KCa1.1 channels can be activated by voltage alone, but coincident calcium entry shifts the voltage dependence of activation of KCa1.1 channels into a physiologically relevant range [2], [35]–[36]. To further test whether calcium influx through Cav3.2 channels could provide a sufficient increase of [Ca]_i_ to mediate a shift in KCa1.1 voltage dependence, cells were held at −100 mV and stepped to voltages ranging from −70 to +40 mV in the presence or absence of coexpressed Cav3.2 channels. The current density-voltage relationship for KCa1.1 activation was then examined with or without a pre-pulse to −30 mV to activate Cav3 calcium influx. When KCa1.1 channels were expressed in isolation, step commands activated whole-cell current at +20 mV, with a steady but slight increase in current with depolarizing steps (*n* = 14) (**Fig. 1** ***C***). In contrast, coexpressing Cav3.2 channels substantially left-shifted the voltage for KCa1.1 activation, with whole-cell KCa1.1 current activation now at approximately −30 mV (−50 mV shift), with a substantial increase in the slope of the I–V relationship (*n* = 13) (**Fig. 1** ***C***). Coexpressing the non-calcium conducting Cav3.2^pm^ with KCa1.1 (*n* = 10) produced an I–V relationship not significantly different from that of KCa1.1 expressed alone (**Fig. 1** ***C***). Furthermore, at a defined intracellular concentration of 1 µM calcium, KCa1.1 activation was similar in the absence and the presence of the pore mutant, suggesting that the mere presence of Cav3.2 did not allosterically alter KCa1.1 activation (data not shown).

KCa1.1 peak current closely paralleled the amount of calcium influx through Cav3.2 channels. In **Figure 1** ***D***, KCa1.1 current recorded in cells coexpressing Cav3.2 channels is plotted as a ratio of current evoked at +40 mV (P2/P1) when the 50 ms prepulse was stepped in 10 mV increments from −90 to +20 mV. The normalized peak Cav3.2 current evoked during each of the prepulse commands is overlaid for comparison. These tests revealed that an initial activation of Cav3.2 current at −60 mV was reflected in a measureable KCa1.1 current evoked by the test pulse to +40 mV (*n* = 10). Moreover, the magnitude of KCa1.1 current tested in this manner closely followed the voltage dependence and magnitude of Cav3.2 calcium influx over the full I–V relationship (**Fig. 1** ***D***). Indeed, coplotting Cav3 and KCa1.1 current at all voltages indicated a correlation coefficient of *r* = 0.836 (*n* = 10). We further examined the time course of KCa1.1 activation by Cav3.2-mediated calcium influx by increasing the amount of time between the Cav3.2 prepulse to −30 mV and the P2 step. These tests showed that the effects of Cav3.2 activation on KCa1.1 current amplitude were greatest in the first 50 ms following the Ca_v_3.2 pre-pulse and then decreased over a period of about 500 ms after Cav3.2 activation (**Fig. 1** ***E***).

**Figure 2:**
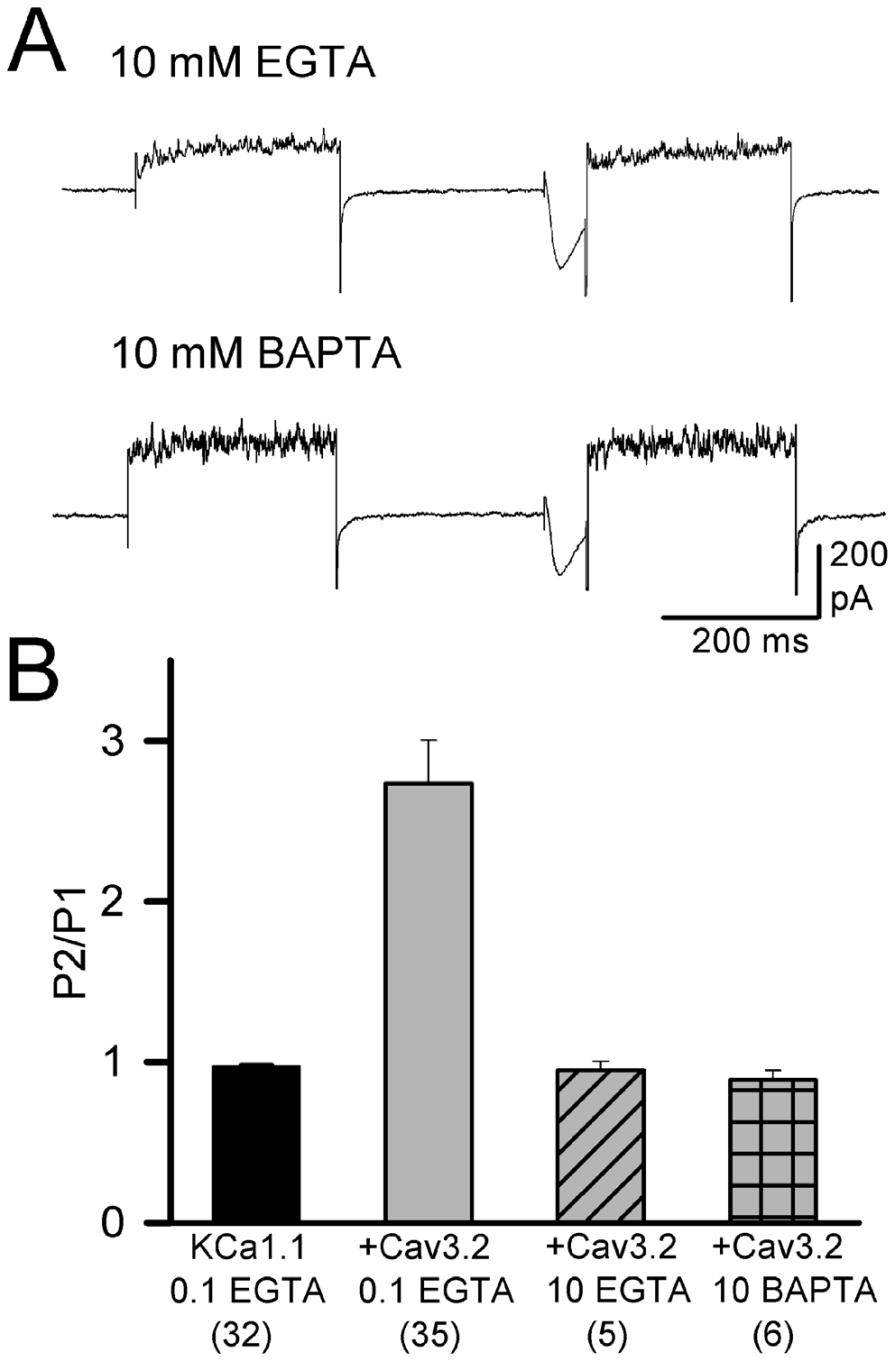
Effects of intracellular calcium buffering on KCa1.1 channel recruitment by Cav3 calcium influx. *A*, Shown are whole-cell recordings from transiently transfected tsA-201 cells, demonstrating the effects of intracellular calcium buffering by 10 mM of EGTA or BAPTA on KCa1.1 current when coexpressed with Cav3.2 channels. *B*, Bar plots indicating the fold change in peak KCa1.1 current (P2/P1) under the indicated conditions of internal calcium chelation when coexpressed with Cav3.2 channels. Calcium entry via Ca_v_3.2 channels increases KCa1.1 channel activity in the presence of internal 0.1 mM EGTA or 0.1 mM BAPTA, but not in the presence of 10 mM of either calcium chelator, or with alternate expression of the nonconducting Ca_v_3.2^pm^. Sample sizes are shown in brackets.

We finally coexpressed the two channels and supplemented the internal recording solution with either 10 mM EGTA or BAPTA, and then examined the ability of co-expressed Cav3.2 channels to activate KCa1.1 current. The inclusion of either calcium buffer blocked the increase in the P2/P1 ratio of KCa1.1 amplitude in all cells examined here (EGTA, *P* = 1.00, *n* = 5; BAPTA, *P* = 0.706, *n* = 6) (**Fig. 2**). Additional tests using 5 mM EGTA in the internal solution established that even this lower concentration of buffer was sufficient to block the increase in the P2/P1 ratio (*n* = 4, data not shown).

### Cav3.2 Channels Coimmunoprecipitate and have Multiple Sites of Interaction with KCa1.1

**Figure 3:**
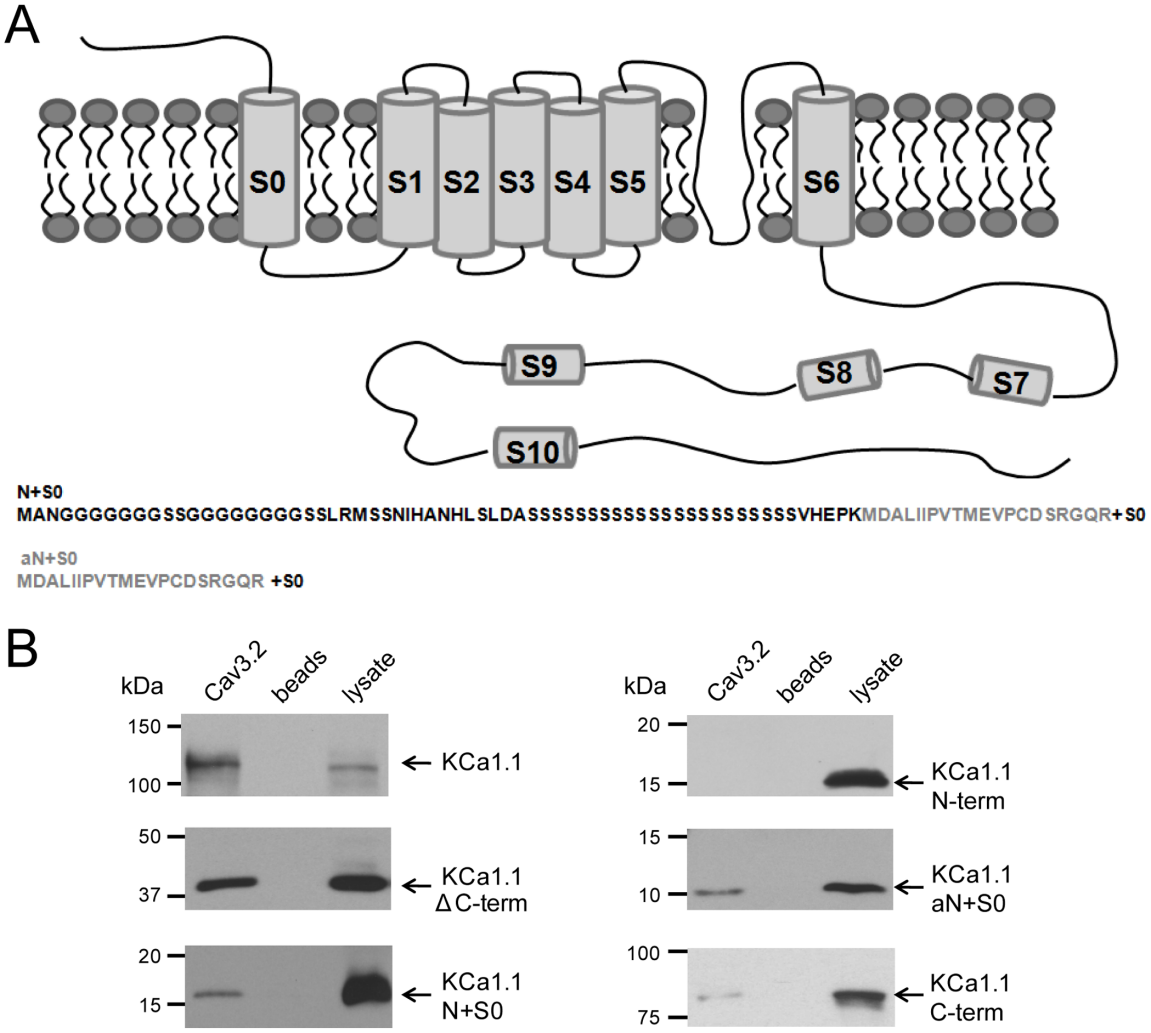
Ca_v_3.2 channels interact with the N-terminal transmembrane region of KCa1.1 channels. *A*, A schematic drawing of the α-subunit of KCa1.1 with the N-terminal segment. Two alternate N-terminal splice variant sequences are shown below the diagram (N+S0, aN+S0) with S0 designating the transmembrane component of the N-terminal region. Region of overlap between KCa1.1 N+S0 and aN+S0 are shown in *grey shading*. Constructs tested include full length KCa1.1, KCa1.1 N+S0, KCa1.1 aN+S0, KCa1.1 N-terminus, KCa1.1 without C-terminus (KCa1.1ΔC-term), and KCa1.1 C-terminus (*n* = 4). *B*, *Left column*, Western blots from lysates of tSA-201 cells indicating that Ca_v_3.2 protein coimmunoprecipitates with full length KCa1.1, KCa1.1ΔC-term, or KCa1.1 N+S0. *Right column*, Cav3.2 channels coimmunoprecipitate with KCa1.1 aN+S0 but not with the KCa1.1 N-terminus. A weak binding was also observed with KCa1.1 C-terminus and Cav3.2. In both panels B and C the first lane corresponds to an immunoprecipitation conducted with a polyclonal Cav3.2 antibody. In the second lane, the precipitating antibody was omitted (i.e., bead only control), and the third lane corresponds to tsA-201 cell lysate. In each case, Western blots were probed with an anti-myc antibody to detect myc-tagged KCa1.1 channels or their fragments. Sample sizes are shown in brackets.

KCa1.1 channels exhibit a 7 transmembrane structure that includes an S0 segment and external N-terminus (**Fig. 3** ***A***). To determine if Cav3.2 channels associate with KCa1.1 at the molecular level we coexpressed the α-subunits of Cav3.2 and KCa1.1 in tsA-201 cells, and were able to coimmunoprecipitate the proteins (**Fig. 3** ***B***). Since these data were obtained with only the α-subunits, it indicates that the interaction does not require β subunits. Consistent with this, coexpressing either β2 or β4 subunits with KCa1.1 and Cav3.2 did not disrupt coimmunoprecipitation between Cav3.2 and KCa1.1 (data not shown).

To further identify the sites of interaction we generated a number of KCa1.1 constructs that contained a myc-tag and co-expressed them with Cav3.2 channels in tsA-201 cells. We then performed a series of coimmunoprecipitation assays to identify which regions associate with full length Cav3.2 channels. We assessed the potential role of the cytoplasmic C-terminus, the external N-terminus, two alternate N-terminal splice variant sequences in conjunction with the S0 transmembrane region of KCa1.1 channels, and the S0–S1 linker. We found that Cav3.2 protein was able to coimmunoprecipitate with full length KCa1.1 and with KCa1.1 channels missing the entire C-terminus (**Fig. 3** ***B***). The latter finding was somewhat surprising given that the KCa1.1 C-terminus contains multiple protein interaction sites shown to interact with other binding partners [37]–[38]. A weak band was detected with the isolated C-terminus of KCa1.1 (visible only after prolonged exposure times), indicating that Cav3.2 may also interact with the C-terminus of KCa1.1, but perhaps with low affinity. However, we detected robust coimmunoprecipitation between Cav3.2 and the KCa1.1 N-terminus containing the S0 transmembrane segment (**Fig. 3** ***B***). By comparison, no coimmunoprecipitation was detected between Cav3.2 and the S0–S1 internal linker (data not shown). To determine whether the N-terminus or the S0 transmembrane segment was the element responsible for coimmunoprecipitation, we took advantage of a naturally occurring short N-terminal splice variant of KCa1.1 [39]. The short extracellular N-terminal fragment along with the S0 segment was able to coimmunoprecipitate, whereas a construct comprised of only the longer extracellular N-terminus (but lacking the S0 segment) did not interact with Cav3.2 (**Fig. 3** ***B***).

These data provide support for an interaction between Cav3.2 and the transmembrane S0 segment of KCa1.1. We were unable to test whether there was a direct interaction between Cav3.2 and S0 alone, as an S0 construct did not express in tsA-201 cells. However, an involvement of the KCa1.1 S0 segment is supported by the observation that two different N-terminal splice variants fused to S0 interacted in a similar manner with Cav3.2, whereas the N-terminal segment alone failed to coimmunoprecipitate. The corresponding site for interaction with Cav3.2 channels also appears to be a transmembrane region, as we failed to pull down KCa1.1 with any of the cytoplasmic regions of Cav3.2 (N-terminus, I–II, II–III, III–IV linkers, C-terminus) in a separate set of experiments (*n* = 6).

### Cav3-KCa1.1 Association in situ

**Figure 4:**
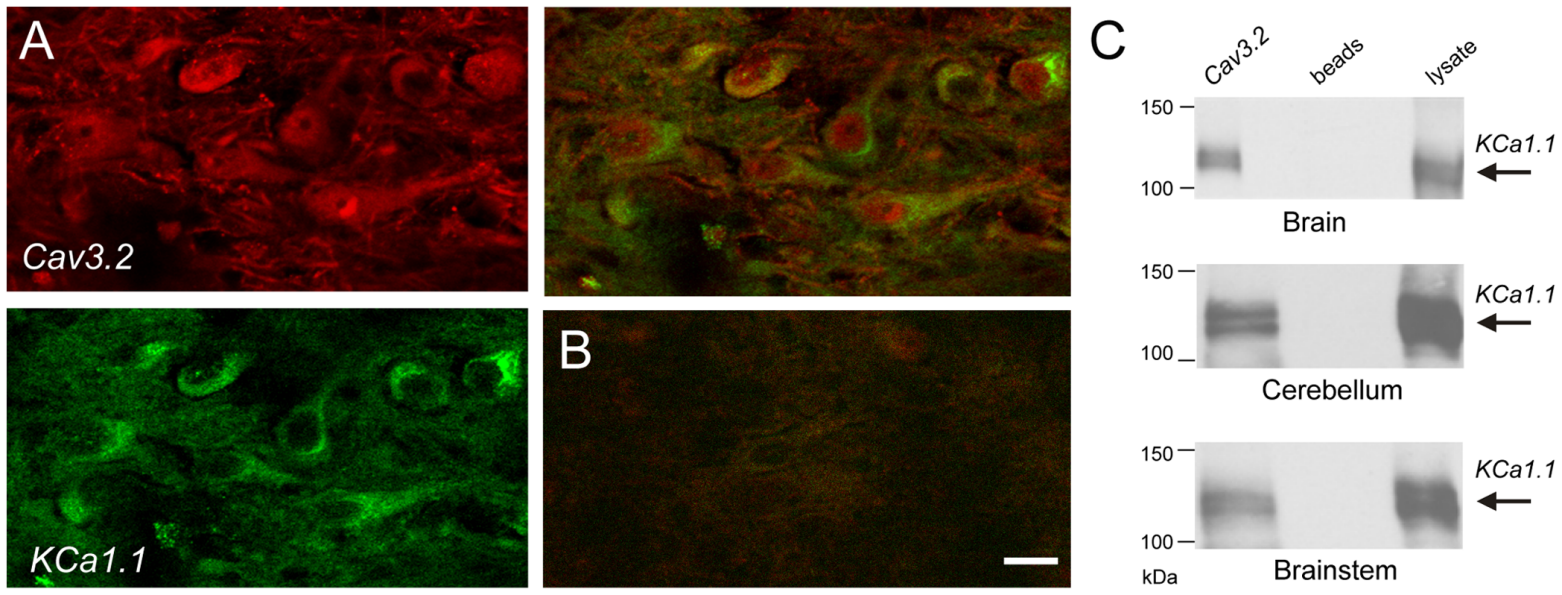
Cav3.2 and KCa1.1 channels associate in rat brain. *A,* Dual label immunocytochemistry confirms Cav3.2 and KCa1.1 protein expression in MVN cells with a similar distribution pattern at the level of the soma and at least the proximal dendritic region. *B,* Control image upon omission of primary antibodies. *C,* Western blots showing coimmunoprecipitation of Cav3.2 and KCa1.1 protein from lysates of rat brain (*n* = 4), cerebellum (*n* = 6), and brain stem (*n* = 5), with a corresponding label for KCa1.1 in lysates from each region. In lane 1, channel complexes were immunoprecipitated using the Cav3.2 antibody, in lane 2 the precipitating antibody was omitted, and lane 3 corresponds to lysate. All Western blots were probed with anti-KCa1.1. Scale bar in (*A, B*) = 20 µm.

The rat MVN is known to contain both GABAergic and non-GABAergic cells that express KCa1.1 channels that control spike output [23], [40]–[42]. KCa1.1 channels in MVN cells were also shown to be sensitive to block by low Ni^2+^ or amiloride application, implying a Cav3 calcium-mediated activation [23]. We thus chose MVN cells as a representative neuronal subtype to further examine the relationship between Cav3 (T-type) current and KCa1.1 channels. To determine whether Cav3 and KCa1.1 channels are codistributed in intact tissue we first conducted dual labeling immunocytochemistry in the rat MVN. Immunocytochemical tests revealed immunolabel for both Cav3.2 and KCa1.1 protein in MVN cells in terms of a diffuse label that shared a distribution at the level of both the soma and at least the proximal extent of dendrites (**Fig. 4** ***A***). Controls consisting of omission of primary antibodies were negative (**Fig. 4** ***B***). As commonly found for light microscopic examination of neuronal ion channels, distinguishing a dual immunolabel at the level of the plasma membrane per se was more difficult to discern despite consistent electrophysiological evidence for Cav3.2 and KCa1.1 currents (see below). In some cases colocalization of the proteins was suggested by a shift to yellow label in superimposed images (**Fig. 4** ***A***). We can thus state that immunocytochemistry confirmed the presence of Cav3.2 and KCa1.1 channel immunolabel in MVN cells, and over the same relative subcellular regions.

To more directly analyze the degree of association between Cav3.2 and KCa1.1 we carried out tests for coimmunoprecipitation in lysates of rat whole brain, cerebellum, and pontine regions. In each case KCa1.1 protein was found to coimmunoprecipitate with Cav3.2 protein at the predicted molecular weight, as supported by detecting a band for KCa1.1 at the same molecular weight in lysates but not control conditions (**Fig. 4** ***C*** and **Fig. S2**). Immunocytochemistry and protein biochemistry thus provide support for coexpression and a close association between Cav3.2 and KCa1.1 channels in the pontine region and other areas of the brain.

We then used patch clamp recordings in *in vitro* tissue slices from P12–P16 rats to examine if Cav3 calcium influx is sufficient to activate KCa1.1 in MVN cells.

### Cav3 Calcium Current

T-type calcium current can derive from any of three different isoforms of the Cav3 family (Cav3.1, Cav3.2, Cav3.3) [43], with both mRNA and protein for each of the Cav3 isoforms reported in MVN cells [44]–[47]. However, there are currently no direct recordings of LVA T-type calcium current in MVN cells. We thus recorded calcium current in the presence of 30 µM Cd^2+^ to block HVA calcium channels (including R-type channels) (see [20], [48]–[49]), a concentration that does not affect Cav3 current [20], [50]–[51]. Additional channel blockers were external TTX (200 nM – 1 µM) (Na^+^), 100 nM apamin (KCa2.x), 2 mM CsCl (HCN), 5 mM 4-AP (Kv3, Kv4), and internal 100 nM TRAM-34 (potential KCa3.1) [20], [40]–[42], [47], [52]_._ The Cav3-mediated component of whole-cell current was then further identified by applying 1 µM mibefradil, or alternatively 300 µM Ni^2+^, a concentration that corresponds to near the reported IC_50_ for Cav3.1 and Cav3.3 calcium channels expressed in tsA-201 cells, and well beyond that for Cav3.2 [32], [53].

**Figure 5:**
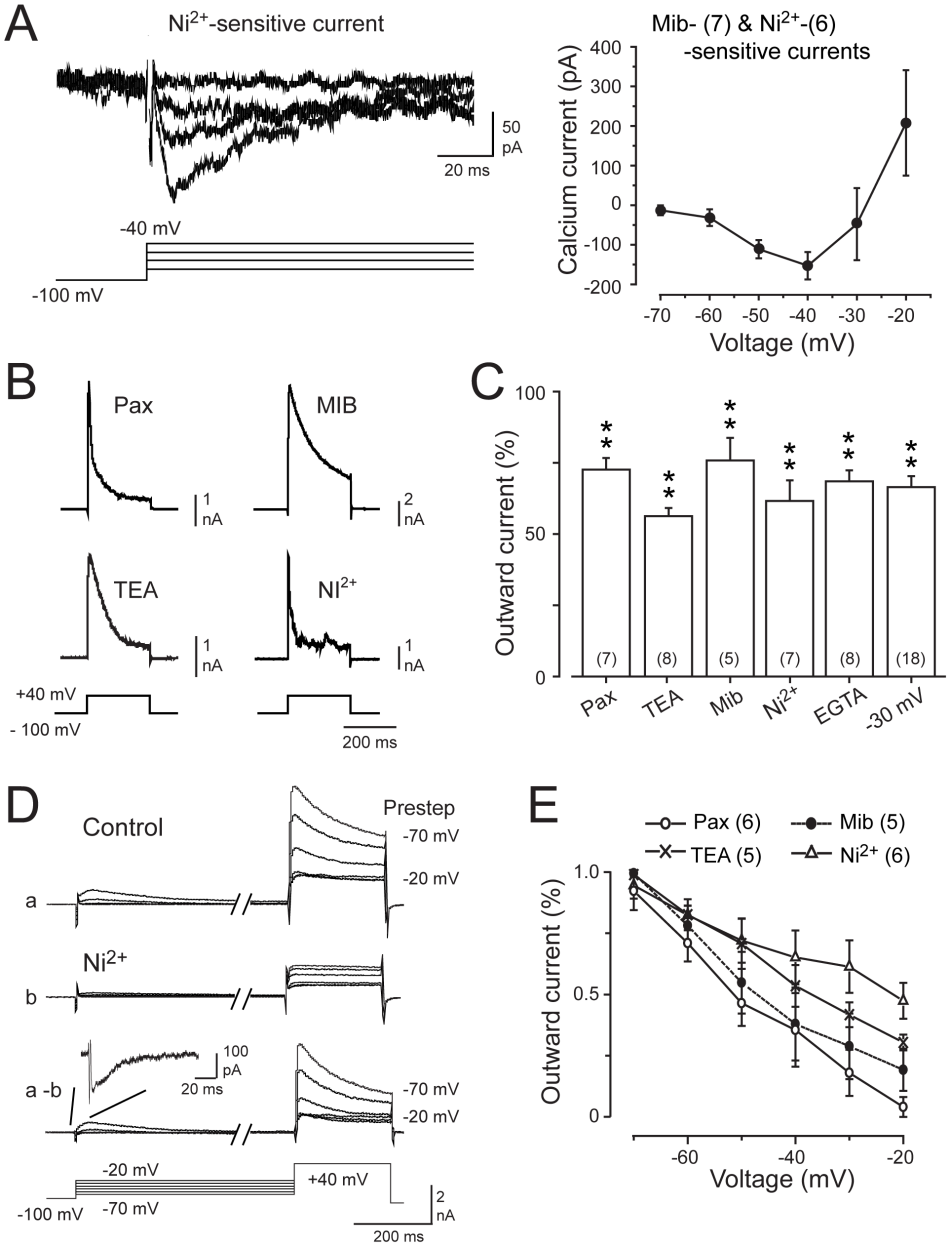
KCa1.1 channels are regulated by Cav3 calcium influx in MVN neurons. *A,* Whole-cell recording of Ni^2+^ (300 µM) -sensitive Cav3 calcium current in the presence of 30 µM Cd^2+^ to block HVA calcium channels. At *right* is the current-voltage relationship for 1 µM mibefradil (Mib)- or Ni^2+^-sensitive currents (data pooled), revealing a LVA inward calcium current that peaks at −40 mV, and progressive activation of outward current at higher membrane voltages. *B,* Representative recordings of outward current sensitive to 1 µM paxilline (Pax) or 1 mM TEA to block KCa1.1 channels, or 1 µM mibefradil or 300 µM Ni^2+^ to block Cav3 channels reveal a similar rapid peak and decaying outward current. *C,* Bar plots of peak outward current evoked by a step from −100 mV to +40 mV. The average result of applying each of 1 µM mibefradil, 300 µM Ni^2+^, 1 mM TEA, 1 µM paxilline, or 10 mM internal EGTA is compared to the extent of KCa1.1 block by inactivating Cav3 channels by a prestep to −30 mV. Mean values were statistically different from original control values, but not from each other (one-way ANOVA F(5,47) = 1.63, *P* = 0.17). *D*, Representative outward current evoked by a step from −100 mV to +40 mV when preceded by preconditioning steps from −70 mV to −20 mV to evoke Cav3 channel inactivation. Shown are traces in control medium (*a*), after perfusing 300 µM Ni^2+^ (*b*), and difference currents (*a–b*). *Inset* shows an expanded view of the Cav3 current activated by an initial prestep to −30 mV. *E,* Mean plots of the voltage-dependence of outward current evoked from −100 mV to the indicated potentials and sensitive to each of the indicated blockers. Sample sizes in (*A*, *C* and *E*) are shown in brackets and capacitance artefacts in (*A*) were reduced digitally for display purposes.

Step commands were delivered from a holding potential of −100 mV over a range of −70 mV to −20 mV and either mibefradil or Ni^2+^ applied to identify the Cav3 channel-mediated component of evoked current. Given the similarity of the results data were pooled for analysis. Subtracting test from control records revealed a fast activating inward current of up to −164 pA peak amplitude that was fast inactivating (τ = 23.4±4.7 ms at −30 mV, *n* = 14) in all MVN cells examined with mibefradil or Ni^2+^ (**Fig. 5** ***A***). The I–V relationship for the transient inward current exhibited initial activation above −60 mV and peaked at ∼−40 mV (**Fig. 5** ***A***). Whole-cell current on I–V plots then transitioned to net outward by ∼−20 mV, as expected for subsequent activation of calcium- and voltage-dependent KCa1.1 current. These recordings then establish that a LVA calcium current with properties consistent with Cav3 calcium channels can be recorded in MVN cells.

*KCa1.1 current:* KCa1.1 current was isolated in all experiments by perfusing TTX (200 nM – 1 µM), 100 nM apamin, 2 mM CsCl, 5 mM 4-AP, and 100 nM internal TRAM-34 [20], [40]–[42], [47], [52]_._ Paxilline (1 µM) was applied as an established KCa1.1 blocker in MVN cells [42], or TEA (1 mM) given the high sensitivity of KCa1.1 channels to this drug (IC_50_ 80–330 µM) [52], and a block of all other TEA-sensitive currents with 5 mM 4-AP. As indicated above, 30 µM Cd^2+^ was present in all experiments to prevent calcium conductance through HVA calcium channels [20], [48]–[49]. We note that a unique aspect of KCa1.1 regulation in MVN cells is that repeated membrane hyperpolarizations over a 5 min period reduce KCa1.1 amplitude by decreasing [Ca]_i_ and the extent of CaMKII-mediated phosphorylation [7], [54]–[55]. We thus expect that the use of a −100 mV holding potential during voltage clamp recordings will reduce [Ca]_i_ and the available KCa1.1 current. Despite these considerations, we found ample KCa1.1 current for experimental measurement in all cells examined.

Steps from −100 mV to +40 mV evoked a large amplitude outward current in all cells examined, with no significant run-down during whole-cell recordings. Application of 1 µM paxilline (*n* = 7) or 1 mM TEA (*n* = 8) to isolate the KCa1.1-mediated component revealed a fast activating current that decayed rapidly over the time course of a 250 ms test pulse (**Fig. 5** ***B***). As reported for other cells [28], [56]–[57], we found some variability in the extent of decrease of KCa1.1 current during the pulse. This variability could potentially reflect a voltage-dependent inactivation process [5], the expression pattern of ß subunits [30]–[31], [58]–[59], or non-ideal space clamp conditions in MVN cells with intact dendritic processes. Since the primary interest was to measure net KCa1.1 current magnitude (as compared to kinetics) we calculated total current (area, pA·ms) and converted to total charge (pC) to compare currents evoked in control and test conditions for a test pulse to +40 mV. In this manner comparable results were obtained between measurements, with more extensive analysis of KCa1.1 voltage-dependent properties assessed in tsA-201 cells (above). Using this approach we found that 1 µM paxilline reduced the total outward current compared to control by 27.4±4.1% (*n* = 7, *P* = 0.007 signed-rank) while TEA reduced the total outward current by 47.2±2.8% (*n* = 8, *P* = 0.007 signed-rank).

### Cav3 Channel Activation of KCa1.1 Current

To determine the ability for Cav3 calcium influx to activate KCa1.1 channels we perfused either 1 µM mibefradil (*n* = 5) or 300 µM Ni^2+^ (*n* = 7) for a step command from −100 mV to +40 mV. Calculating the difference between control and test records revealed outward currents that again exhibited a fast transient peak and rapid decay over a 250 ms command pulse; not unlike that found for paxilline and TEA-sensitive currents (**Fig. 5** ***B and C***). The extent of calcium-dependent potassium current was further identified by dialyzing 10 mM EGTA through the pipette, which decreased total outward current evoked by a command pulse to +40 mV by 33.7±3.8% in 8 of 12 recordings (*n* = 8, *P* = 0.007 signed rank) (**Fig. 5** ***C***). The reason that EGTA did not reduce current in all cells was not determined, but is not expected to reflect a lack of KCa1.1 current because subsequent addition of TEA blocked the outward current by 32.5±10% in the remaining 4 cells (see Discussion). Finally, we compared the results of pharmacological tests to the extent of outward current reduced by inactivating Cav3 channels using a preconditioning step to −30 mV (3 sec), once again reducing outward current by 33.5±3.8% (*n* = 18) (**Fig. 5** ***C***). Importantly, the degree of KCa1.1 current reduced upon inactivating Cav3 channels was not significantly different from the reductions induced by paxilline, TEA, mibefradil, Ni^2+^, or EGTA (one-way ANOVA F(5,47) = 1.63, *P* = 0.17).

If the outward current evoked under these conditions reflects activation by Cav3 channel-mediated calcium influx one would predict that potassium current would exhibit similar voltage dependence to that of Cav3 channels. We thus preceded a test pulse from −100 mV to +40 mV with a family of preconditioning pulses from −70 mV to −20 mV (3 sec) to determine the effects of Cav3 inactivation on outward current (**Fig. 5** ***D***). These tests confirmed the initial activation of a fast inward current at the beginning of the preconditioning pulse that steadily inactivated as the amplitude of the prepulse increased. The preconditioning steps were also associated with a graded decrease in evoked outward current by the test pulse to +40 mV (**Fig. 5** ***D***). Subsequent perfusion of mibefradil (*n* = 5) or Ni^2+^ (*n* = 6) to isolate the Cav3-mediated component of evoked responses blocked the decaying component of outward current during the +40 mV step (**Fig. 5** ***D***), exhibiting a similar voltage dependent reduction over the full set of preconditioning steps (**Fig. 5** ***E***). Finally, repeating these tests for perfusion of either paxilline (*n* = 6) or TEA (*n* = 5) isolated KCa1.1 current that again exhibited a comparable voltage-dependent reduction as that found after Cav3 channel blockade (**Fig. 5** ***E***). Together these data indicate that the characteristics of KCa1.1 current were consistent with those expected for initial activation by a low voltage and inactivating Cav3 calcium current.

### Low Voltage Regulation of KCa1.1

The coupling between Cav3 and KCa1.1 channels in MVN cells was highly effective in the suprathreshold range, as tested using a single +40 mV voltage command pulse. A second question is the potential for Cav3 channels to trigger KCa1.1 current in the low voltage range of membrane potential. Some evidence for this capability can be detected in the preconditioning step inactivation protocol of **Figure 5** ***D and E*** **,** in that a difference in the degree of KCa1.1 activation was apparent for preconditioning steps between −70 mV and −60 mV. To further test this we used a depolarizing ramp command from −100 mV to −30 mV over 500 ms to test the ability for Cav3 current to activate KCa1.1 current in the same recording conditions (in the presence of external TTX, CsCl, and Cd^2+^ as above).

**Figure 6:**
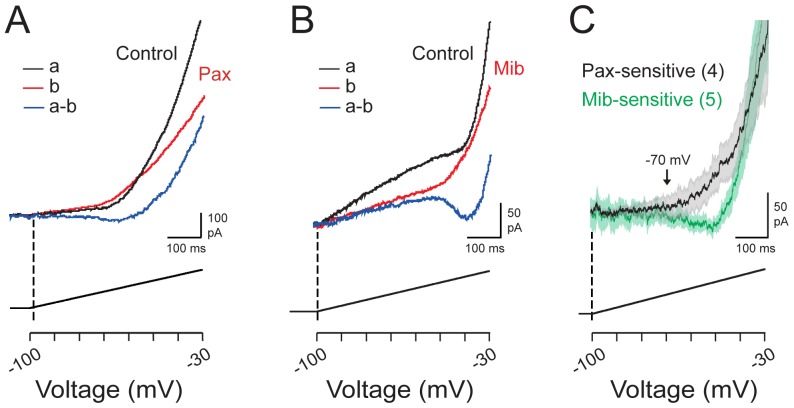
The Cav3-KCa1.1 interaction can activate KCa1.1 in the subthreshold voltage range. Representative whole-cell currents in MVN cells during a ramp command from −100 mV to −30 mV in the presence of 30 µM Cd^2+^ to block HVA calcium channels. *Dashed lines* indicate onset of the voltage ramp command. The relative time during the ramp at which different voltages are attained are shown on a voltage scale below. *A,* Outward current apparent in control recordings (*black* trace) is reduced by the KCa1.1 channel blocker paxilline (1 µM) (*red* trace) and isolated below as a difference current (*a-b, blue* trace). *B,* Recordings from a separate cell under the same conditions as in (*A*). Perfusing 1 µM mibefradil (Mib, *red* trace) blocks both inward and outward currents apparent in control recordings (*black* trace), with the difference current indicated below (*a-b*, *blue* trace). *C*, Superimposed average records of currents sensitive to paxilline (*black* trace) or mibefradil (*green* trace). Current recordings in (*C*) are average traces with SEM shown by shaded regions. Sample sizes are shown in brackets.

Ramp commands elicited a gradually rising outward current associated with the voltage ramp stimulus, and a superimposed inward current with a low voltage for onset that increased rapidly in downward slope above ∼−60 mV (*n* = 23) (**Fig. 6**). Inward current then transitioned to a more steeply rising outward current to the end of the command pulse. Perfusion of 1 µM paxilline during a ramp command selectively reduced the outward current activated in this low voltage range in 4 out of 11 cells (**Fig. 6** ***A and C***). In a separate set of experiments 1 µM mibefradil also reduced the inward transient and the outward current during ramps from −100 mV to −30 mV in 6 out of 12 cells (**Fig. 6** ***B and C*** **)**. Calculating the difference between control and test records in these cases revealed the activation of inward current even ∼−70 mV, with a peak of typically ∼50 pA at ∼−45 mV (**Fig. 6** ***C***). The inward current was then followed by a rise in outward current that transitioned rapidly upwards at ∼−20 mV. Superimposing mean currents sensitive to either paxilline or mibefradil indicated a close correspondence between the initial onset of Cav3 calcium current to the onset of paxilline-sensitive outward current (**Fig. 6** ***C***).

**Figure 7:**
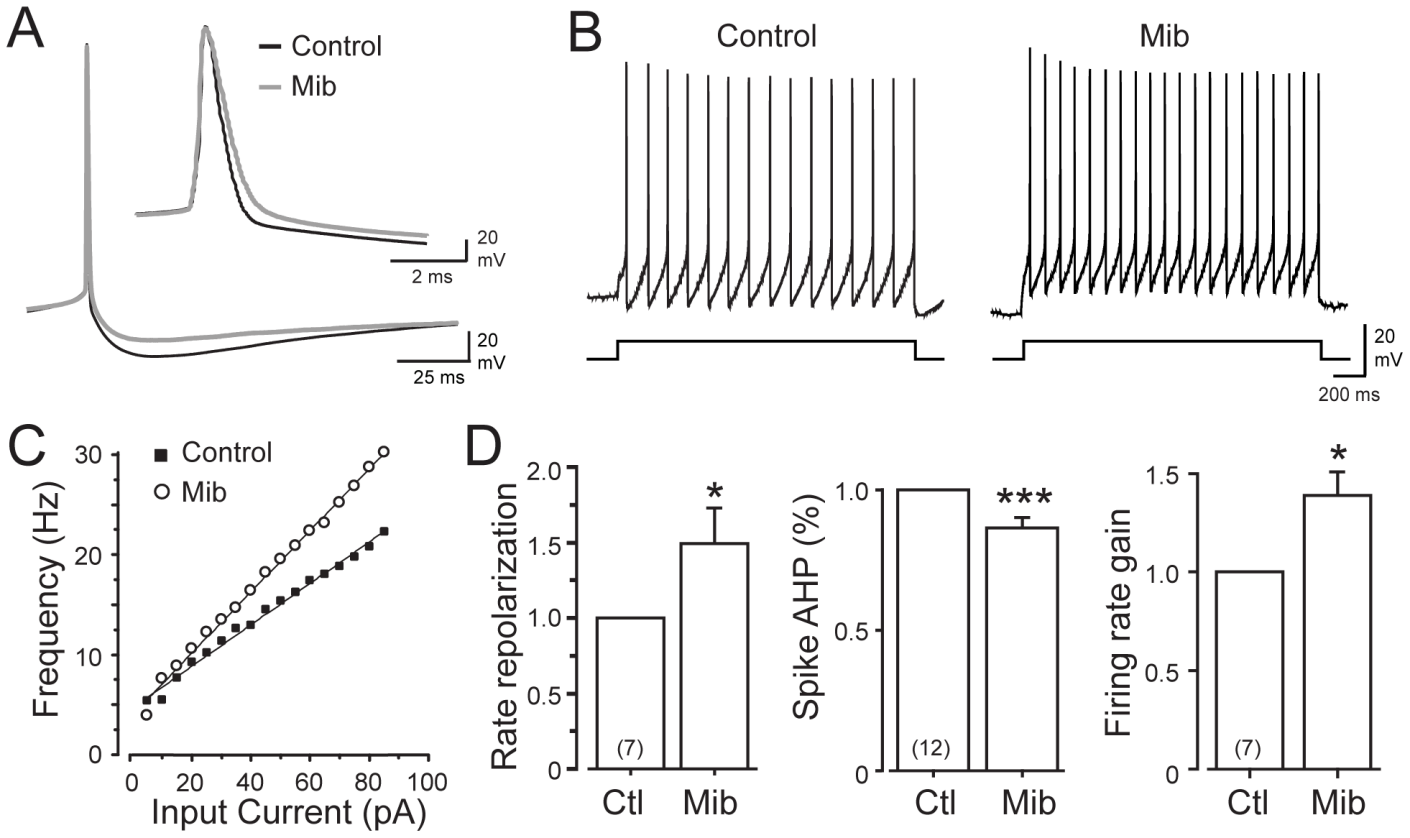
Cav3 calcium activation of KCa1.1 modulates repolarizing responses and gain of spike firing. *A,* Representative superimposed recordings of spike discharge before and after perfusion of 1 µM mibefradil (Mib) to block the Cav3-KCa1.1 interaction. Mibefradil slows that rate of repolarization (*inset*) and reduces the AHP. *B,* Representative examples of spike firing during square wave current pulse injections before and after 1 µM mibefradil showing an increase in the rate of firing. *C*, Current-frequency plot for the cell in (*B*) reveals an increase in the gain of firing frequency in mibefradil. *D*, Bar plots of the mean rate of spike repolarization, AHP amplitude, and firing rate gain on current-frequency plots before and after mibefradil. Membrane potential was set through bias current injection to ∼−74 mV to subdue baseline tonic firing of MVN cells and spike properties were measured near threshold firing level evoked by square wave current injections. Sample sizes are shown in brackets in (*D*).

To gauge the physiological effects of Cav3 activation of KCa1.1 channels we recorded the membrane potential and spike response of MVN cells under current clamp conditions. For these experiments cells were held by bias current injection to −75 mV. In one set of tests a ramp current injection of 500 ms was applied to slowly raise a cell from a hyperpolarized level and through to spike discharge in the manner used for voltage clamp recordings. The amplitude of ramp current injection was adjusted before mibefradil application to elicit one spike and kept constant for the rest of the recording. The first spike evoked by a given ramp was used for latency and threshold analyses. Application of 1 µM mibefradil during a ramp depolarization did not significantly change spike threshold (control, −52.7±2.1 mV; test, −53.9±2.5 mV, *n* = 10, *P* = 0.20), the latency to spike discharge (*n* = 10, *P = *0.39), or the slope of the depolarization during the ramp (*n* = 10, *P* = 0.92). A second set of tests used square wave current pulse injections (1 sec duration) to construct current-frequency plots of spike output. Again we again found no significant difference in spike threshold upon mibefradil application (control, −52.8±1.7 mV; test, −55.1±1.8 mV, *n* = 12, *P* = 0.09). However, during repetitive spike trains mibefradil significantly increased the gain of firing in 7 of 12 cells, increasing from 26+4 spikes/100 pA to 36+7 spikes/100 pA (*n = *7, *P* = 0.02) (**Fig. 7** ***B–D***). The rate of spike repolarization also slowed in the presence of mibefradil by a mean factor of 1.55X (*P* = 0.015) in 7/12 cells (**Fig. 7** ***A and D***). Notably, the effects of mibefradil on the rate of repolarization or firing rate gain were not clearly correlated, suggesting cell-specific effects within the MVN cell population. However, there was a highly significant decline in the amplitude of the AHP in all cells by 13.5+3.2% (*n* = 12, *P* = 0.0002) (**Fig. 7** ***A and D***).

Together these results confirm that Cav3-mediated calcium influx can activate KCa1.1 current in the low voltage range of ∼−50 mV, well below that of most voltage-gated potassium channels that activate above −40 mV [52]. However, the greatest influence of the Cav3-KCa1.1 interaction in MVN cells was detected under current-clamp conditions as a (cell-specific) change in repolarization or gain of firing frequency, revealing activation of Cav3 current during the spike response.

## Discussion

The ability of calcium-gated potassium channels to control cell excitability depends on a calcium source to trigger their activation. Previous work indicates that specific isoforms of HVA calcium channels can activate KCa1.1 channels to contribute to spike repolarization and generating AHPs [2], [6], [9]–[10], [55], [60]–[61]. The association between HVA calcium and KCa1.1 potassium channels can extend to the molecular level, as shown through coimmunoprecipitation between KCa1.1 channels and Cav1.2 and Cav1.3 (L-type), Cav2.1 (P/Q) and Cav2.2 (N-type) channels [12]–[13]. This offers a distinct advantage in that cells can use different HVA calcium channel isoforms to selectively activate KCa1.1 channels depending on spike output requirements [2], [8], [11], [14].

The current study used tsA-201 cells and MVN neurons to investigate KCa1.1 current activation under conditions designed to restrict calcium influx to LVA Cav3 calcium channels. MVN cells were chosen as a representative neuronal cell type since KCa1.1 channels are expressed in all MVN cell types characterized to date [42], [47], and provide a significant component of outward current driving the AHP [7], [40], [54]–[55]. Activation of KCa1.1 current during spike discharge has also been carefully analyzed in MVN cells [7], [40]–[42], [55], although the specific relationship between LVA calcium current and KCa1.1 activation was not determined. We now show that KCa1.1 channels form a molecular and functional association with Cav3 calcium channels that allows the unique voltage-dependent properties of Cav3 channels to dictate the nature of KCa1.1 channel activation in both the LVA and HVA range of membrane potential.

### KCa1.1 Channel Activation by Cav3 Calcium Influx

It has been shown that KCa1.1 current can follow the voltage dependence and time course (kinetics) of specific HVA calcium channel isoforms [15], and over the more extended voltage range provided by Cav1.3 calcium channels [9]–[10]. We found that the molecular association between Cav3 and KCa1.1 channels also allow several aspects of KCa1.1 activation in tsA-201 and MVN cells to parallel the voltage-dependent properties of Cav3 calcium channels. In MVN cells Cav3 calcium influx activated KCa1.1 current from as low as −70 mV for ramp stimuli as well as during square wave steps from −100 mV to +40 mV, and that these were sensitive to block by low concentrations of mibefradil or Ni^2+^. The amplitude of KCa1.1 current paralleled the amplitude of Cav3 calcium current over a full I–V relationship in tsA-201 cells (**Fig. 1** ***D***) and was closely related to the extent of Cav3 calcium channel inactivation induced by depolarizing presteps in MVN cells (**Fig. 5** ***E***). In fact, there was a strong correlation between Cav3 and KCa1.1 current recorded in tsA-201 cells (*r* = 0.86), where both currents initially activated at ∼−60 mV, peaked at ∼−45 mV, with a rapid increase in outward current above ∼−50 mV. By comparison, initial activation of KCa1.1 current by Cav2.1 (P/Q-type) calcium current occurs near −30 mV and peaks at 0 mV, and for Cav1.2 (L-type) calcium influx at 0 mV and peaks at +30 mV [15]. Thus, the Cav3-KCa1.1 complex identified here provides an entirely different voltage range for KCa1.1 activation than that for HVA calcium channels.

### Cav3-mediated KCa1.1 Activation in situ

The Cav3-KCa1.1 interaction could be reproduced in tsA-201 cells upon coexpressing only the α-subunits of Cav3.2 and KCa1.1, indicating that these are sufficient to account for the principal interaction between the two channels. However, a key difference between the Cav3-KCa1.1 interaction recorded in MVN and tsA-201 cells was the degree of KCa1.1 activation by a single command pulse. In tsA-201 cells a single pulse from −100 mV to +40 mV recruited very little KCa1.1 current while in MVN cells a step of this size consistently recruited sufficient Cav3 calcium current to activate KCa1.1 channels. Once Cav3 current was provided in tsA-201 cells by a prestep command KCa1.1 amplitude was substantially increased, with the pharmacology and voltage dependence of the interaction essentially equivalent between tsA-201 and MVN cells.

These data indicate the existence of factors in MVN cells that increase the sensitivity of the Cav3-KCa1.1 complex over that in tsA-201 cells that could arise through the association of β or other auxiliary subunits with the KCa1.1 channel [58]–[59], [62]–[63] in MVN cells, which were not expressed in tsA-201 cells. However, the identity and composition of potential auxiliary subunits associated with KCa1.1 channels in MVN cells has not been established. The greater sensitivity of KCa1.1 channels to Cav3 calcium influx in MVN cells might also reflect a difference in the localization of the complex that restricts the diffusion for calcium, thereby increasing the effectiveness of calcium influx. Immunocytochemistry indicated that Cav3.2 and KCa1.1 immunolabel is distributed over the same relative areas of soma and proximal dendrites of MVN cells, but this does not guarantee formation of a complex capable of promoting a direct interaction between Cav3 calcium influx and KCa1.1 activation. Regardless, a Cav3-KCa1.1 functional association at the membrane level was consistently demonstrated in terms of reduction in KCa1.1 current by mibefradil, Ni^2+^, or a preconditioning pulse to inactivate Cav3 channels.

### Molecular Basis for a Cav3.2-KCa1.1 Interaction

An interaction between HVA calcium and KCa1.1 potassium channels has been well established, although the specific site(s) of interaction at the molecular level have not been identified [12]–[13]. Here we show coimmunoprecipitation of Cav3.2 and KCa1.1 channels from rat whole brain, cerebellum, or brain stem (**Fig. 4** **, Fig. S2**). We note that coimmunoprecipitation was also obtained between Cav3.1 and Cav3.3 channels with KCa1.1 from lysates of rat brain, although the specifics of these interactions were not further pursued here (data not shown).

It was previously suggested that the site of KCa1.1 interaction with HVA calcium channels occurs through a transmembrane region [2]. We were able to narrow down the area of Cav3.2 interaction to two sites on KCa1.1 channels. The most robust interaction occurred through the S0 transmembrane segment of the KCa1.1 N-terminal region, one of several sites important for the interaction of β subunits with KCa1.1 channels [64]–[65]. It is possible that the two proteins compete for the same site of interaction, although we found that the expression of either β2 or β4 subunits (the most commonly found β isoforms in the brain) did not prevent coimmunoprecipitation between KCa1.1 and Cav3.2. Finally, a weak interaction occurs between Cav3.2 and the KCa1.1 C-terminus, a structural component that encompasses approximately 2/3 of the entire KCa1.1 amino acid sequence and has been indicated in a number of protein-protein interactions [66]–[67].

### Interaction between Cav3 and KCa1.1 Channels

It has been established that an association between HVA calcium and KCa1.1 channels allow an interaction at the level of a nanodomain, suggesting a spatial proximity of <50 nm [12], [68]. Here we show that Cav3 and KCa1.1 channels are also part of a macromolecular signaling complex as detected through coimmunoprecipitation. Yet unlike HVA channels, the Cav3 mediated activation of KCa1.1 channels was sensitive to intracellular EGTA in tsA-201 cells and some MVN cells. This EGTA sensitivity is more consistent with an interaction typically defined as falling within a microdomain [68]–[70], implying a greater distance between the two channels (i.e. >200 nm) than would perhaps be expected from a direct molecular association.

So how can these seemingly disparate findings be reconciled? Unlike HVA calcium channels, Cav3 channels show a comparatively small open probability (0.25 Cav3 vs 0.80 Cav2.2), small single channel conductance (1.7 pS at 2 mM [Ca]_o_), and rapid inactivation (within 100 ms) [71]–[72]. As a result, each individual T-type calcium channel may not be sufficient to increase [Ca]_i_ to the level (i.e., 10 µM [2]) required to reliably activate KCa1.1 channels. Hence, the concerted action of multiple Cav3 channels may be needed to activate KCa1.1. To explore this possibility we constructed a computational model (see **Methods**) where potassium channel activation was determined by simulated calcium diffusion through 10 hemispherical compartments of increasing radii (20–200 nm, 20 nm increments). Cav3 and KCa1.1 channel properties were modeled according to those reported for physiological levels of [Ca]_o_ and voltage-dependence and kinetics of human Cav3.2 channels expressed in tsA-201 cells.

**Figure 8:**
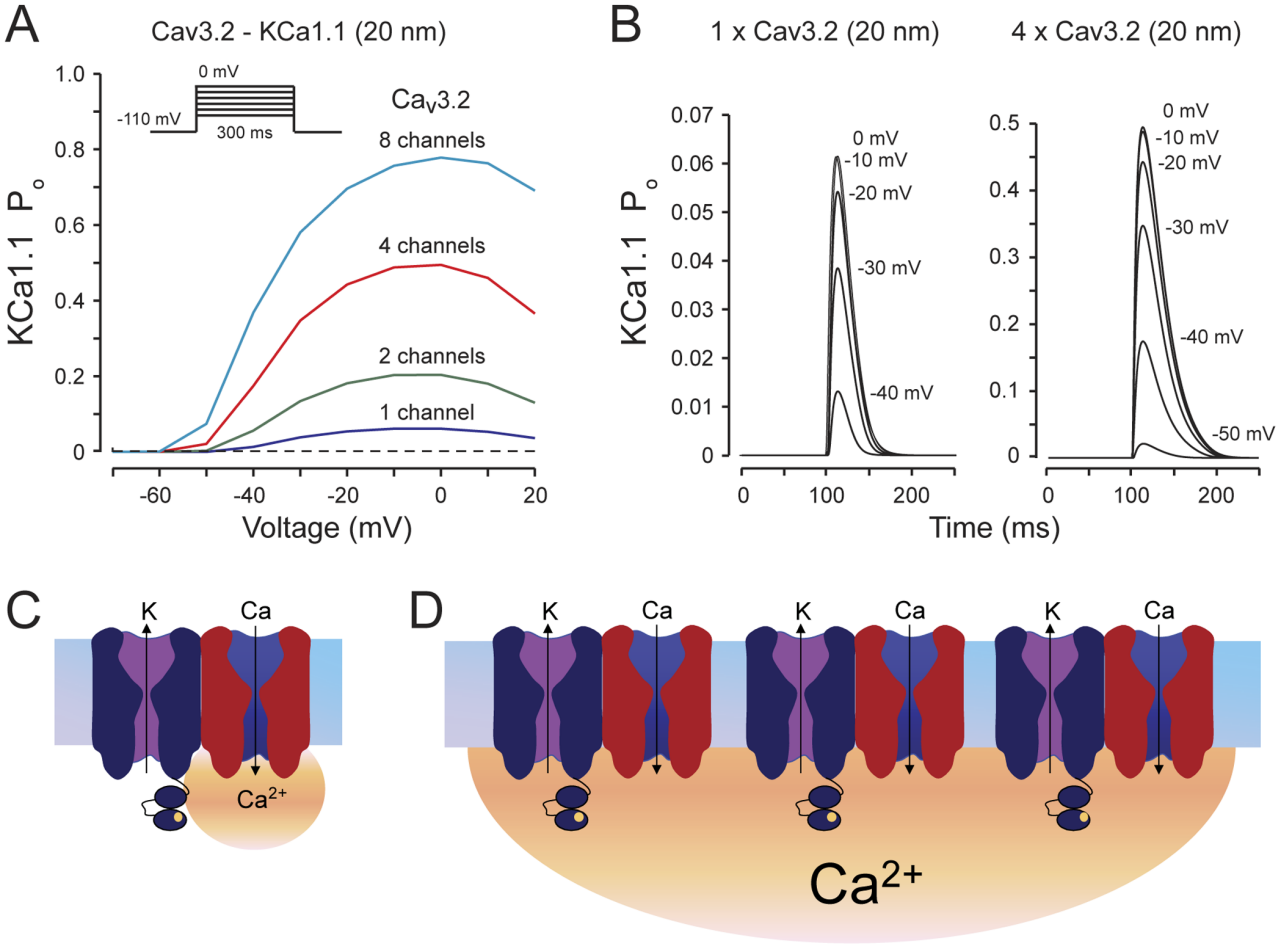
Model of Cav3 calcium channel domain and interaction with KCa1.1 channels. A calcium diffusion model is used to simulate the interaction between a Cav3 calcium source and a KCa1.1 channel located within its nanodomain (20 nm). Cav3.2 and KCa1.1 properties are based on reported values in the presence of physiological levels of [Ca]_o_. *A,* Activation curves for the KCa1.1 model based on the increase of [Ca]_i_ when 1, 2, 4, or 8 Cav3 channels are positioned within 20 nm (see Methods). A single Cav3 channel causes no activation of a KCa1.1 channel below −30 mV and only minimal activation at high voltages. Activation of KCa1.1 at low voltages (−50 mV) is only attained with 4 or 8 Cav3 channels. *B,* Activation time courses during voltage steps as in (*A*) for models with either 1 or 4 Cav3 channels positioned within 20 nm of KCa1.1. KCa1.1 activation is transient, tracking the Cav3-mediated calcium influx, but with much higher probability when 4 Cav3 channels contribute. *C, D,* Schematic diagrams of a proposed model for Cav3 activation of KCa1.1 channels. In the case of a single Cav3 channel (*C*) a small calcium domain provides only a low probability of generating the increase in [Ca]_i_ necessary to activate KCa1.1 channels. By comparison, summation of calcium domains generated by several neighboring Cav3/KCa1.1 channel complexes (*D*) is sufficient to activate KCa1.1. Such a contribution of more distant Cav3 channels would then be consistent with the EGTA sensitivity observed in our experiments.

Under these conditions we found that a single Cav3 channel would be virtually incapable of activating KCa1.1 channel even when positioned at 20 nm distance as expected from a molecular interaction between the two channels (**Fig. 8** ***A, B***). Even at such close distances summation of the intracellular calcium domains of at least 4 Cav3 channels was required to reach the threshold for KCa1.1 activation. Hence, although Cav3 and KCa1.1 channels form protein complexes, calcium influx through multiple neighboring channel complexes is necessary to create a large enough calcium microdomain to activate KCa1.1 (as depicted schematically in **Figs. 8** ***C, D***). We recognize that this model represents only a first approximation of the factors that will control calcium domains and the numbers of Cav3 channels needed; however, the requirement for the combined action of multiple Cav3-KCa1.1 complexes perhaps spaced over greater distances would account for the observed EGTA sensitivity. In this context, forming an association at the molecular level may ensure the correct colocalization of these channels in subcellular regions where KCa1.1 current is required, and perhaps serve to enhance KCa1.1 channel forward trafficking as has been reported for TRPC6/KCa1.1 complexes [73–55].

The requirement for a summation of calcium domains has also been previously observed with calcium-dependent inactivation (CDI) of L-type and P/Q-type calcium channels - a process that relies in both cases on calmodulin molecules that are physically associated with these channels [76]. In the case of L-type channels, CDI involves calcium interactions with the high affinity calcium binding sites on calmodulin, and hence an individual L-type channel is able to trigger its own calcium inactivation [77]. In the case of P/Q-type channels CDI involves the low affinity calcium binding site of calmodulin, and thus requires an ensemble of P/Q-type channels to raise calcium to sufficiently high levels to permit CDI. With L-type channels CDI is EGTA-insensitive, while CDI of P/Q channels is blocked by EGTA (akin to EGTA blocking the Cav3- KCa1.1 interaction).

Altogether, these considerations suggest that the amount of calcium entering through a tethered calcium channel, together with the calcium sensitivity of the partner KCa1.1 channel may lie at the root of the differential EGTA sensitivity of various Cav3-K_Ca_ complexes.

### Physiological Role of Cav3-KCa1.1 Activation

The expression studies in tsA-201 cells and voltage clamp analysis provide direct biophysical evidence for KCa1.1 activation from as low as ∼−70 mV and coincident with Cav3 current activation (**Fig. 6** ***C***). Despite this, detecting the outcome of KCa1.1 current on membrane excitability more negative than −50 mV proved more difficult under current clamp conditions. The sharpest increase in KCa1.1 activation using voltage ramp commands occurred in the low voltage range of ∼−50 mV. While this voltage range is apparently just beyond a point that could regulate spike threshold (mean of −53 mV) under our conditions, mibefradil consistently uncovered a contribution by the Cav3-KCa1.1 activation to the AHP following spike discharge, with ∼60% of cells exhibiting additional effects on the rate of spike repolarization and firing rate gain. These data indicate an activation of the Cav3-KCa1.1 complex as membrane voltage moves from a low voltage of −50 mV and through the generation of spike discharge. Indeed, the sharp increase in KCa1.1 activation near −50 mV likely reflects the synergistic interplay between [Ca]_i_ and membrane voltage on KCa1.1 activation. A similar activation of Cav3 calcium channels during spike discharge was reported in cerebellar Purkinje cells, where it was estimated that ∼50% of the calcium current during sodium spike discharge is carried by Cav3 channels followed by shaping of the interspike membrane potential [78]. A relatively low voltage activation of KCa1.1 channels by Cav1.3-mediated calcium influx has also been shown in chromaffin cells, where KCa1.1 current contributes to AHPs and modifying the interspike interval [10] in a manner similar to that mediated by Cav3 channels in MVN cells.

The cell-specific differences detected here on repolarizing responses is consistent with a known differential expression pattern of potassium channels in MVN cells [40], [42], [47] and the extent of coupling to Cav3 calcium channels. In this regard, our results with mibefradil align closely with those previously reported in MVN cells for perfusion of Ni^2+^ or amiloride, where these blockers produced cell-specific effects on rates of spike repolarization, AHP, and firing rate gain [23]. Thus, Ni^2+^ or amiloride increased firing rate gain by 22–80% [23], not unlike a range of 5–88% (mean of 37%) increase in gain observed here with mibefradil application. It is also important to note that a substantial component of the AHP remained after mibefradil application, confirming previous work that established a significant role for HVA calcium channels (particularly Cav2.2 (N-type) channels) in activating KCa1.1 channels in MVN cells [23]. Interestingly, the role of Cav3 and Cav2.2 channels in regulating a slower component AHP in MVN cells resembles that reported for deep cerebellar neurons [50], [79]–[80], a homologous cell type that is also in direct receipt of Purkinje cell inhibitory input. Previous work on the association between HVA calcium and KCa1.1 channels further indicates that coexpression of these subunits does not guarantee association as a complex, in that HVA calcium and KCa1.1 channels can be recorded in isolation or as a functional complex within individual cells [11].

In summary, the current work reveals that Cav3 calcium channels form a molecular association with the transmembrane domain of the N-terminal region of KCa1.1 potassium channels. As a result, Cav3 activation of KCa1.1 channels extends the voltage range for KCa1.1 activation beyond that provided by HVA calcium channels. This is important in that Cav3 channels provide a reliable source of voltage-gated calcium influx near spike threshold, with key control over spike responses following a membrane hyperpolarization [51], [81]–[82]. Given the widespread expression pattern of both channel subtypes we expect that the Cav3-KCa1.1 complex identified here will be functional and important to regulating membrane excitability in a wide range of neuronal cell types.

## Supporting Information

Figure S1 **KCa1.1 co-expression does not change Ca_v_3.2 current density or voltage-dependent properties.** Whole-cell patch clamp recordings from transiently transfected tsA-201 cells. *A*, Currents were evoked in 10 mV increments from a holding potential of −90 mV. There is no significant change in peak current density or maximum conductance between conditions where Ca_v_3.2 is expressed alone (*black circles*) or co-expressed with KCa1.1 (*grey circles*). *B,* Cav3 currents were evoked using a pre-pulse protocol with an initial step to −30 mV to maximally activate Ca_v_3.2 followed by a test pulse, which ranged from −110 mV in 5 mV increments to 0 mV. The half inactivation potential (V_h_) and half activation potential (V_a_) were calculated using a modified Boltzmann equation. Sample sizes are shown in brackets.
(TIF)

Figure S2 **Western blots showing coimmunoprecipitation of Cav3.2 and KCa1.1 channel from lysates of rat brain (** ***n*** **= 3), cerebellum (** ***n*** **= 3), and brain stem (** ***n*** **= 3), with a corresponding label for Cav3.2 in lysates from each region.** Each lysate, corresponding coimmunoprecipitation and bead control were ran together on the same membrane however Cav3.2 in the lysate was only visible after longer exposure (30 sec compared to 5 min respectively).
(TIF)
