# Supplementary figures and images for: Low Voltage Activation of KCa1.1 Current by Cav3-KCa1.1 Complexes
Source: PLoS One. 2013 Apr 23;8(4):e61844. doi: 10.1371/journal.pone.0061844 (PMC3633930; doi:10.1371/journal.pone.0061844)

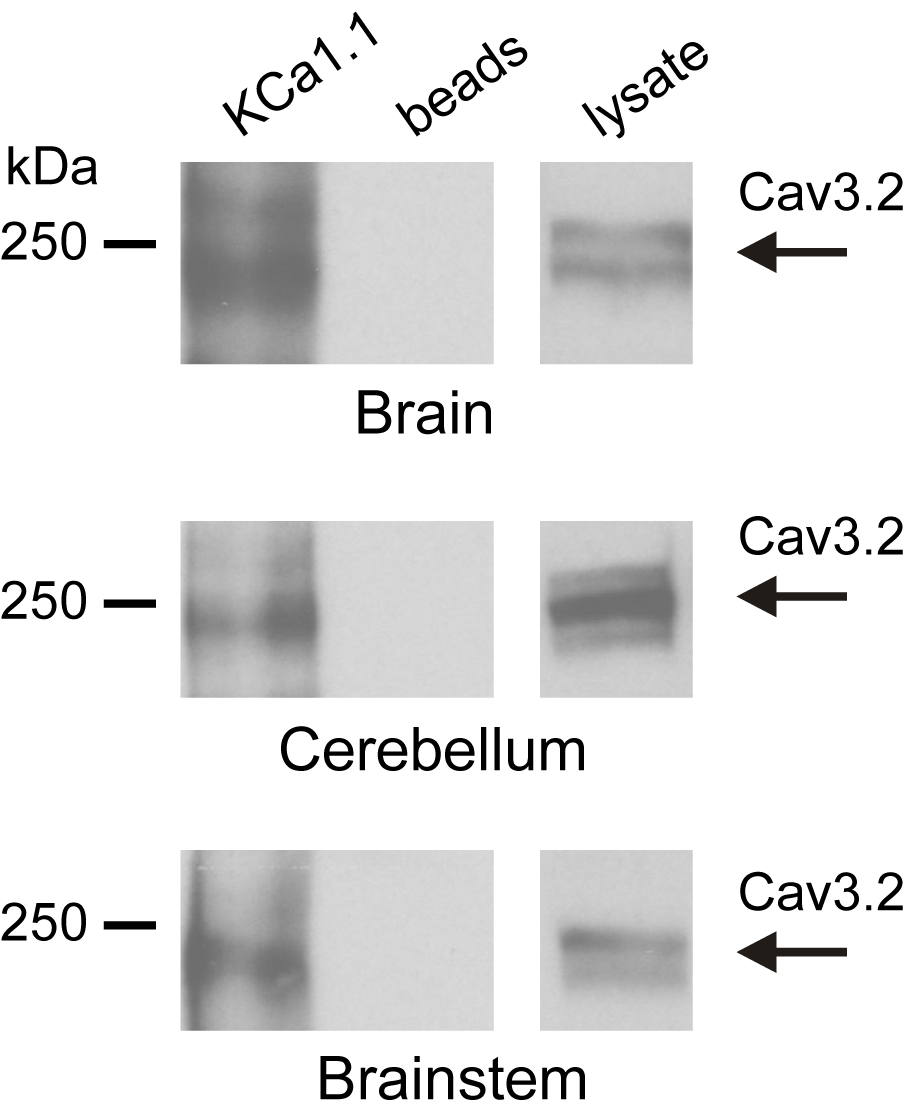

Supplement: Figure S2 — Western blots showing coimmunoprecipitation of Cav3.2 and KCa1.1 channel from lysates of rat brain ( n = 3), cerebellum ( n = 3), and brain stem ( n = 3), with a corresponding label for Cav3.2 in lysates from each region. Each lysate, corresponding coimmunoprecipitation and bead control were ran together on the same membrane however Cav3.2 in the lysate was only visible after longer exposure (30 sec compared to 5 min respectively). (TIF) [file pone.0061844.s002.tif]
